# Supplementary material for: A tree-planting decision support tool for urban heat mitigation
Source: PLoS One. 2020 Oct 8;15(10):e0224959. doi: 10.1371/journal.pone.0224959 (PMC7544061; doi:10.1371/journal.pone.0224959)
Supplement: S1 Methods — (DOCX) [file pone.0224959.s001.docx]

**S1 Methods**. **Heat Vulnerability Index data sources and calculations.**

Socio-demographic data were extracted from the American Communities Survey and National Land Cover Database at census tract level following the variable selections of  Nayak et al. [1] from the New York State Heat Vulnerability Index (HVI) (S1 Table). The ACS 2009-2013 5-year estimates were used for all ACS variables and joined to census 2010 tracts. Housing density was calculated using the weighted estimate divided by census tract area.

In order to process the NLCD data, we used the “raster to polygon” tool in ArcGIS Pro, and selected the attributes of polygons to identify the “high building intensity areas” and “open undeveloped areas,” which we then intersected with census tracts to calculate the percentage of land with each land cover characteristic by census tract. Rotated Principal Components Analysis on variables (varimax rotation) was then used to retain factors following Nayak et al. [1] wherein “eigenvalues greater than 1, a clear break in values in the scree test, and the percentage of variance explained by the factors” [2] as well as interpretability (a determination of shared, underlying meaning of the grouping of variables).” We calculated principal components factors using 2011 factor weights for both time points. We multiplied each variable by these factor weightings (S2 Table). Finally, we standardized the components to have a mean of 0 and a standard deviation of 1, assigned categories (1-6) indicating relative vulnerabilities (from least to most vulnerable), and then summed the components for overall HVI score.

**References:**

1. Nayak SG, Shrestha S, Kinney PL, Ross Z, Sheridan SC, Pantea CI, et al. Development of a heat vulnerability index for New York State. Public Health. 2018;161: 127–137. doi:10.1016/j.puhe.2017.09.006
2. Reid CE, O’Neill MS, Gronlund CJ, Brines SJ, Brown DG, Diez-Roux AV, et al. Mapping Community Determinants of Heat Vulnerability. Environmental Health Perspectives. 2009;117: 1730–1736. doi:[10.1289/ehp.0900683](https://doi.org/10.1289/ehp.0900683)
