# Supplementary material for: A tree-planting decision support tool for urban heat mitigation
Source: PLoS One. 2020 Oct 8;15(10):e0224959. doi: 10.1371/journal.pone.0224959 (PMC7544061; doi:10.1371/journal.pone.0224959)
Supplement: S1 Table — (DOCX) [file pone.0224959.s002.docx]

**S1 Table. Data sources and variables used in Heat Vulnerability Index calculation.**

| **Source** | **Time Period** | **Variables** |
| --- | --- | --- |
| US Census American Community Survey | 2009-2013 5-year estimate | •        Percentage population  •        that is **Hispanic**  •        that is **Black**  •        that is **foreign born**  •        who **speak English less than ‘very well’**  •        with income **below poverty level**  •        **over 65 years of age**  •        **over 65 years of age** and **living alone**  •        (18–64 years) that has a **disability***  •        (18–64 years) that are **unemployed**  •        Percentage houses **built before 1980**  •        Density of **housing units per square mile** |
| National Land Cover Database | 2011 | •        Percentage land with **high building intensity areas**  •        Percentage land that consists of **open undeveloped areas** |
