# Supplementary material for: A tree-planting decision support tool for urban heat mitigation
Source: PLoS One. 2020 Oct 8;15(10):e0224959. doi: 10.1371/journal.pone.0224959 (PMC7544061; doi:10.1371/journal.pone.0224959)
Supplement: S2 Table — (DOCX) [file pone.0224959.s003.docx]

**S2 Table. Principal component factor weights used to calculate Heat Vulnerability Index**.

| **Rotated Factor Pattern** | **Socio-demographic component** | **Environmental/ urbanicity component** | **Social isolation/ elderly component** |
| --- | --- | --- | --- |
| Hispanic | **0.74*** | 0.34 | -0.12 |
| Non-English speaking | **0.73*** | 0.50 | -0.13 |
| Below poverty line | **0.79*** | 0.36 | 0.00 |
| With a disability | **0.84*** | 0.04 | 0.12 |
| Unemployed | **0.73*** | 0.07 | -0.12 |
| Black | **0.58*** | 0.26 | -0.14 |
| Open, undeveloped land | -0.26 | **-0.82*** | -0.01 |
| High building intensity | 0.33 | **0.77*** | -0.09 |
| Foreign born | 0.36 | **0.69*** | -0.17 |
| Older homes | 0.16 | **0.61*** | 0.11 |
| Housing density | 0.01 | **0.78*** | -0.14 |
| ≥ 65  years old | -0.22 | -0.19 | **0.87*** |
| ≥ 65  years old & living alone | 0.03 | 0.05 | **0.93*** |
