# Supplementary material for: A tree-planting decision support tool for urban heat mitigation
Source: PLoS One. 2020 Oct 8;15(10):e0224959. doi: 10.1371/journal.pone.0224959 (PMC7544061; doi:10.1371/journal.pone.0224959)
Supplement: S3 Table — Land surface temperature was calculated using remotely-sensed midmorning temperatures during the months of July-August. Heat Vulnerability Index (HVI) was derived using demographic data from the American Communities Survey (2009–2013 estimate) and the 2011 National Land Cover Database. Current and potential tree canopy cover were estimated using the 2014 Boston Land Cover dataset from the University of Vermont Spatial Analysis Lab. (DOCX) [file pone.0224959.s004.docx]

**S3 Table. Heat Vulnerability Index, summer land surface temperature, and potential tree canopy for all Boston census tracts.** Land surface temperature was calculated using remotely-sensed midmorning temperatures during the months of July-August [1,2]. Heat Vulnerability Index (HVI) was derived using demographic data from the American Communities Survey (2009-2013 estimate) [3] and the 2011 National Land Cover Database [4]. Current and potential tree canopy cover were estimated using the 2014 Boston Land Cover dataset from the University of Vermont Spatial Analysis Lab [5].

| **Census tract** | **HVI** | **Land surface temperature** | **Current tree canopy cover (%)** | **Potential tree canopy cover (%)** | **Neighborhood** |
| --- | --- | --- | --- | --- | --- |
| 25025000100 | 18 | 37.41 | 18.6 | 14.1 | Brighton |
| 25025000201 | 16 | 34.39 | 25.8 | 16.7 | Brighton |
| 25025000202 | 16 | 34.39 | 23.6 | 17.7 | Brighton |
| 25025000301 | 17 | 34.39 | 31.7 | 14.8 | Brighton |
| 25025000302 | 16 | 34.39 | 37.7 | 18.5 | Brighton |
| 25025000401 | 20 | 37.97 | 23.8 | 12.8 | Brighton |
| 25025000402 | 16 | 34.39 | 32.6 | 21.3 | Brighton |
| 25025000502 | 16 | 37.97 | 28.5 | 18.9 | Brighton |
| 25025000503 | 18 | 37.97 | 22.9 | 7.5 | Brighton |
| 25025000504 | 18 | 37.97 | 27.2 | 8.5 | Brighton |
| 25025000601 | 16 | 39.44 | 20.3 | 15.3 | Brighton |
| 25025000602 | 18 | 37.97 | 32.4 | 12.7 | Brighton |
| 25025000701 | 18 | 37.97 | 17.1 | 8 | Brighton |
| 25025000703 | 17 | 40.89 | 9.1 | 3.3 | Allston |
| 25025000704 | 18 | 40.89 | 10.8 | 4.5 | Allston |
| 25025000802 | 17 | 40.89 | 14.8 | 7.8 | Brighton |
| 25025000803 | 14 | 40.89 | 9.1 | 11.8 | Fenway |
| 25025010103 | 13 | 39.48 | 12.5 | 8.1 | Fenway |
| 25025010104 | 17 | 39.48 | 13.7 | 5.8 | Back Bay |
| 25025010203 | 16 | 38.28 | 12.1 | 7 | Fenway |
| 25025010204 | 15 | 39.48 | 12.1 | 4.5 | Fenway |
| 25025010300 | 15 | 38.28 | 17 | 12.4 | Longwood Medical Area |
| 25025010403 | 22 | 38.28 | 8.1 | 3.6 | Back Bay |
| 25025010404 | 19 | 38.28 | 13.4 | 6 | Fenway |
| 25025010405 | 17 | 38.28 | 14.7 | 8.1 | Mission Hill |
| 25025010408 | 18 | 38.4 | 17.5 | 8.6 | Fenway |
| 25025010500 | 18 | 38.28 | 13.1 | 3.3 | Back Bay |
| 25025010600 | 16 | 36.91 | 7.4 | 1.8 | Back Bay |
| 25025010701 | 19 | 37.27 | 21.9 | 2.4 | Back Bay |
| 25025010702 | 16 | 37.27 | 24.1 | 3 | Back Bay |
| 25025010801 | 18 | 37.27 | 16.8 | 3.1 | Back Bay |
| 25025010802 | 19 | 37.27 | 19.7 | 4.3 | Back Bay |
| 25025020101 | 19 | 37.27 | 19.4 | 2 | Back Bay |
| 25025020200 | 18 | 38.22 | 13.3 | 1.7 | West End |
| 25025020301 | 18 | 38.22 | 20.7 | 9.2 | West End |
| 25025020302 | 19 | 35.87 | 12.1 | 5.9 | West End |
| 25025020303 | 15 | 35.87 | 14.3 | 8.1 | North End |
| 25025030100 | 18 | 40.53 | 12.5 | 5.1 | North End |
| 25025030200 | 19 | 40.53 | 3.7 | 2 | North End |
| 25025030300 | 17 | 40.53 | 6.8 | 2.7 | North End |
| 25025030400 | 18 | 40.53 | 6.7 | 3.1 | North End |
| 25025030500 | 15 | 40.53 | 7.1 | 3.9 | North End |
| 25025040100 | 15 | 37.38 | 16.4 | 7.5 | Charlestown |
| 25025040200 | 20 | 37.38 | 20.6 | 7.6 | Charlestown |
| 25025040300 | 17 | 37.38 | 14.7 | 5.5 | Charlestown |
| 25025040401 | 16 | 37.38 | 13 | 13.5 | Charlestown |
| 25025040600 | 14 | 38.39 | 15.2 | 12 | Charlestown |
| 25025040801 | 16 | 37.38 | 11.9 | 8.7 | Charlestown |
| 25025050101 | 21 | 37.97 | 12.2 | 7.3 | East Boston |
| 25025050200 | 21 | 37.97 | 12.2 | 5.9 | East Boston |
| 25025050300 | 18 | 37.97 | 9.5 | 15.2 | East Boston |
| 25025050400 | 21 | 35.67 | 9.5 | 6.2 | East Boston |
| 25025050500 | 21 | 37.62 | 9.8 | 9.3 | East Boston |
| 25025050600 | 21 | 37.62 | 10.6 | 4.4 | East Boston |
| 25025050700 | 21 | 37.62 | 13.4 | 11.2 | East Boston |
| 25025050901 | 20 | 37.24 | 9.1 | 9.7 | East Boston |
| 25025051000 | 18 | 35.95 | 14.7 | 19.9 | East Boston |
| 25025051101 | 19 | 31.15 | 18.7 | 19.2 | East Boston |
| 25025051200 | 19 | 37.62 | 15.4 | 11.7 | East Boston |
| 25025060101 | 16 | 32.58 | 12.1 | 7.8 | South Boston |
| 25025060200 | 16 | 32.58 | 15.4 | 9.7 | South Boston |
| 25025060301 | 16 | 32.58 | 16.4 | 7.5 | South Boston |
| 25025060400 | 17 | 32.58 | 17.3 | 11.1 | South Boston |
| 25025060501 | 15 | 39.02 | 12.2 | 9.8 | South Boston Waterfront |
| 25025060600 | 12 | 39.02 | 4.3 | 4.8 | South Boston Waterfront |
| 25025060700 | 17 | 39.62 | 21.8 | 10.5 | South Boston |
| 25025060800 | 15 | 39.02 | 10.7 | 4.3 | South Boston |
| 25025061000 | 19 | 32.58 | 15.7 | 8.1 | South Boston |
| 25025061101 | 21 | 32.58 | 33.5 | 12.6 | Dorchester |
| 25025061200 | 14 | 32.58 | 6.5 | 5.3 | South End |
| 25025070101 | 14 | 35.87 | 2.9 | 1.7 | Leather District |
| 25025070200 | 18 | 31.21 | 6.6 | 3.3 | Bay Village |
| 25025070300 | 17 | 36.91 | 16.9 | 2.1 | Bay Village |
| 25025070402 | 21 | 37.55 | 11.4 | 2.3 | Bay Village |
| 25025070500 | 18 | 38.61 | 25.1 | 6 | South End |
| 25025070600 | 17 | 38.57 | 27.5 | 2.1 | South End |
| 25025070700 | 17 | 37.91 | 22.3 | 3.3 | South End |
| 25025070800 | 18 | 38.28 | 23 | 6.4 | Roxbury |
| 25025070900 | 19 | 38.57 | 19.2 | 5.7 | Roxbury |
| 25025071101 | 18 | 37.38 | 11.9 | 7 | Roxbury |
| 25025071201 | 16 | 38.61 | 8.6 | 5.3 | South End |
| 25025080100 | 18 | 39.06 | 16.9 | 11.4 | Roxbury |
| 25025080300 | 17 | 38.57 | 23.2 | 13.4 | Roxbury |
| 25025080401 | 17 | 38.57 | 21.1 | 10.9 | Roxbury |
| 25025080500 | 20 | 38.57 | 23.2 | 17 | Roxbury |
| 25025080601 | 15 | 38.28 | 15.6 | 14.1 | Mission Hill |
| 25025080801 | 15 | 38.28 | 17.2 | 14.6 | Mission Hill |
| 25025080900 | 17 | 37.38 | 18.6 | 14.2 | Mission Hill |
| 25025081001 | 19 | 37.38 | 18.5 | 8.7 | Mission Hill |
| 25025081100 | 14 | 36.59 | 31.8 | 11.5 | Jamaica Plain |
| 25025081200 | 19 | 36.59 | 22.5 | 11.9 | Jamaica Plain |
| 25025081300 | 19 | 29.72 | 21.7 | 12.9 | Jamaica Plain |
| 25025081400 | 17 | 37.67 | 31.5 | 14.7 | Jamaica Plain |
| 25025081500 | 18 | 36.12 | 35.3 | 18.1 | Roxbury |
| 25025081700 | 19 | 38.57 | 27.3 | 14.6 | Roxbury |
| 25025081800 | 19 | 37.27 | 24.7 | 15.5 | Roxbury |
| 25025081900 | 20 | 29.72 | 26 | 19.2 | Roxbury |
| 25025082000 | 20 | 35.88 | 25.7 | 16.7 | Roxbury |
| 25025082100 | 21 | 29.72 | 23.1 | 14.8 | Roxbury |
| 25025090100 | 20 | 29.72 | 20.4 | 13 | Roxbury |
| 25025090200 | 18 | 35.64 | 17.2 | 10.2 | Roxbury |
| 25025090300 | 18 | 36.27 | 19.6 | 15.9 | Roxbury |
| 25025090400 | 18 | 35.96 | 21.2 | 18.2 | Roxbury |
| 25025090600 | 18 | 37.97 | 17.7 | 14.9 | Roxbury |
| 25025090700 | 17 | 34.95 | 13.2 | 9.5 | Roxbury |
| 25025090901 | 16 | 32.58 | 17.1 | 14.3 | Dorchester |
| 25025091001 | 17 | 32.58 | 18.1 | 13.6 | Dorchester |
| 25025091100 | 18 | 35.74 | 16.9 | 10.1 | Dorchester |
| 25025091200 | 17 | 35.74 | 24.5 | 10.8 | Dorchester |
| 25025091300 | 18 | 35.96 | 16.7 | 9 | Roxbury |
| 25025091400 | 17 | 36.27 | 26 | 15.5 | Roxbury |
| 25025091500 | 19 | 36.27 | 28.1 | 9.7 | Dorchester |
| 25025091600 | 19 | 36.97 | 20 | 10.7 | Dorchester |
| 25025091700 | 20 | 36.97 | 16.9 | 18 | Dorchester |
| 25025091800 | 19 | 36.97 | 23.9 | 12.4 | Dorchester |
| 25025091900 | 18 | 36.25 | 30.9 | 14.5 | Dorchester |
| 25025092000 | 19 | 36.15 | 20.1 | 12.2 | Dorchester |
| 25025092101 | 18 | 36.32 | 17.3 | 13.2 | Dorchester |
| 25025092200 | 18 | 35.96 | 29.5 | 14.7 | Dorchester |
| 25025092300 | 18 | 35.96 | 23.5 | 13.7 | Dorchester |
| 25025092400 | 17 | 29.72 | 21.2 | 16.7 | Roxbury |
| 25025100100 | 18 | 29.72 | 19.3 | 29.6 | Roxbury |
| 25025100200 | 17 | 35.74 | 26.6 | 16.6 | Mattapan |
| 25025100300 | 18 | 35.96 | 29.3 | 15.8 | Mattapan |
| 25025100400 | 19 | 32.84 | 26.5 | 16.3 | Mattapan |
| 25025100500 | 19 | 32.84 | 25.9 | 14.5 | Dorchester |
| 25025100601 | 18 | 32.84 | 21.9 | 14.4 | Dorchester |
| 25025100603 | 16 | 36.32 | 16.7 | 21.1 | Dorchester |
| 25025100700 | 16 | 32.84 | 17.7 | 22.2 | Dorchester |
| 25025100800 | 18 | 32.84 | 29.6 | 21.6 | Mattapan |
| 25025100900 | 18 | 32.84 | 32.2 | 17.8 | Mattapan |
| 25025101001 | 20 | 34.38 | 41.7 | 14.8 | Roslindale |
| 25025101002 | 21 | 35.74 | 33.1 | 20.7 | Mattapan |
| 25025101101 | 18 | 36.92 | 23.1 | 17.4 | Mattapan |
| 25025101102 | 20 | 35.74 | 23.7 | 18.5 | Mattapan |
| 25025110103 | 16 | 29.72 | 32.9 | 11.6 | Roslindale |
| 25025110201 | 18 | 34.38 | 32 | 15.6 | Roslindale |
| 25025110301 | 17 | 35.29 | 29.7 | 24.4 | Roslindale |
| 25025110401 | 18 | 34.57 | 32.2 | 19.8 | Roslindale |
| 25025110403 | 18 | 34.38 | 37.3 | 20.4 | Roslindale |
| 25025110501 | 16 | 34.57 | 29.2 | 20.4 | Roslindale |
| 25025110502 | 17 | 34.57 | 28 | 17.9 | Roslindale |
| 25025110601 | 16 | 34.57 | 54.6 | 15.3 | Roslindale |
| 25025110607 | 17 | 34.57 | 33.5 | 19.4 | Roslindale |
| 25025120103 | 17 | 34.6 | 39 | 15.4 | Jamaica Plain |
| 25025120104 | 16 | 36.42 | 37.3 | 11.6 | Jamaica Plain |
| 25025120105 | 18 | 34.6 | 58.7 | 17.8 | Jamaica Plain |
| 25025120201 | 17 | 29.72 | 27.2 | 11 | Jamaica Plain |
| 25025120301 | 17 | 29.72 | 29.4 | 12.5 | Jamaica Plain |
| 25025120400 | 16 | 36.42 | 39.1 | 14.2 | Jamaica Plain |
| 25025120500 | 18 | 36.59 | 26.1 | 8.1 | Jamaica Plain |
| 25025120600 | 17 | 36.59 | 35.3 | 12.5 | Jamaica Plain |
| 25025120700 | 17 | 36.59 | 25 | 13 | Jamaica Plain |
| 25025130100 | 16 | 35.92 | 41.9 | 27.5 | West Roxbury |
| 25025130200 | 16 | 34.57 | 32.4 | 20.8 | Roslindale |
| 25025130300 | 16 | 34.57 | 35.5 | 22 | Roslindale |
| 25025130402 | 17 | 35.92 | 29.3 | 21.6 | West Roxbury |
| 25025130404 | 16 | 35.92 | 47.7 | 18.9 | West Roxbury |
| 25025130406 | 19 | 34.68 | 39.8 | 20 | West Roxbury |
| 25025140102 | 17 | 35.91 | 34.2 | 20 | West Roxbury |
| 25025140105 | 18 | 34.38 | 40.4 | 15.5 | Roslindale |
| 25025140106 | 18 | 36.13 | 32.9 | 15.1 | Roslindale |
| 25025140107 | 19 | 35.91 | 34.7 | 20.5 | Hyde Park |
| 25025140201 | 17 | 35.91 | 29.2 | 18.6 | Hyde Park |
| 25025140202 | 18 | 35.91 | 40.8 | 21.7 | Hyde Park |
| 25025140300 | 19 | 34.38 | 31.5 | 17.6 | Hyde Park |
| 25025140400 | 19 | 34.38 | 35.2 | 18.3 | Roslindale |
| 25025980101 | NA | 28.84 | 29.5 | 36.3 | Harbor Islands |
| 25025980300 | NA | 29.72 | 56.9 | 29.9 | Jamaica Plain |
| 25025981100 | 12 | 29.72 | 43.1 | 44.2 | Roslindale |
| 25025981202 | NA | 32.58 | 2.8 | 5.1 | South Boston Waterfront |
| 25025981300 | 13 | 37.24 | 1.3 | 31 | East Boston |
| 25025981800 | 14 | 38.28 | 61.2 | 22.1 | Jamaica Plain |

**Works Cited**

[1] Wang, J. A., L. R. Hutyra, D. Li, and M. A. Friedl, 2017: Gradients of Atmospheric Temperature and Humidity Controlled by Local Urban Land-Use Intensity in Boston. *J. Appl. Meteor. Climatol.*, **56**, 817–831, <https://doi.org/10.1175/JAMC-D-16-0325.1>.

[2] Wang, J.A, 2019: "Land surface temperature and urban heat island effects on air temperature and vapor pressure deficit in Boston, MA", <https://doi.org/10.7910/DVN/J8EDZN>, *Harvard Dataverse,* V1

[3] American Communities Survey. U.S. Census Bureau. 2018.

[4] Homer C, Fry J. The National Land Cover Database. US Geol Surv Fact Sheet. 2012; 1–4.

[5] University of Vermont Spatial Analysis Lab. Boston Land Cover. 2014. Available: https://www.tpl.org/climate-smart-cities–boston
